# Peripheral Nerve Blocks as a Predictor of Nerve Reconstruction Success After Major Limb Amputation

**DOI:** 10.7759/cureus.69458

**Published:** 2024-09-15

**Authors:** Daisy E Martinez, Anthony G DeMartino, Georg J Furtmüller, Khanjan Nagarsheth

**Affiliations:** 1 Vascular Surgery, University of Maryland School of Medicine, Baltimore, USA; 2 Vascular Surgery, University of Maryland Medical Center, Baltimore, USA

**Keywords:** neuroma, peripheral nerve block, postamputation pain, regenerative peripheral nerve interface, targeted muscle reinnervation

## Abstract

Postamputation pain is a spectrum of debilitating sensations that impacts millions of people in the United States. While the development of postamputation pain, including phantom limb pain (PLP), is multifactorial, it has been associated with disorganized axonal sprouting, resulting in a neuroma and subsequent central nervous system changes. Nerve reconstruction surgeries, such as regenerative peripheral nerve interface (RPNI) and targeted muscle reinnervation (TMR), provide transected nerve fibers with proper target organs for reinnervation and have been shown to significantly reduce PLP. This case series aims to describe perioperative peripheral nerve blocks as a diagnostic tool for identifying patients who would benefit from RPNI or TMR. We conducted a retrospective search of patients who underwent major extremity amputation and who received a diagnostic peripheral nerve block before undergoing reconstructive nerve surgery (TMR and/or RPNI). Six patients (58-80 years old) with below-knee amputations (BKA) were examined. All patients experienced a reduction in postamputation pain (PAP), specifically PLP, after a diagnostic peripheral nerve block (PNB). The average time between amputation and revision surgery was approximately two years (Mean: 22.35 months). Following surgical intervention, all patients reported a reduction in PLP episodes after nerve reconstruction surgery. Two patients no longer reported PLP. Ambulation rates also improved following revision (50% vs 83%). PNBs can be used as an effective diagnostic tool to identify patients that will significantly benefit from amputation revisions with TMR or RPNI.

## Introduction

Around 185,000 major limb amputations are performed nationally each year, and nearly 2 million amputees currently live in the United States, with an expected rise to 3.6 million by the year 2050 [1,2]. Major limb amputation often has a significant impact on patients’ functional status and overall quality of life. One negative contributing factor to post-amputation outcomes is the development of postamputation pain (PAP). As many as 85% of patients after major lower extremity amputation experience PAP, which encompasses a spectrum of sensations, including residual limb pain, phantom limb sensations, or phantom limb pain (PLP) alone or in any combination [3-5].

The underlying causes of PAP are often complex, ranging from wound healing issues secondary to ischemia, infection, or mechanical causes to neuropathologic changes within the peripheral and central nervous system [6,7]. The nature of major extremity amputation is unique, as distal nerve stumps and adequate target organs are lost, leading to unsuccessful axonal regeneration and scar formation, which fosters neural inflammation and hyperexcitability [4,8,9]. Frequently, the formation of terminal neuroma at the proximal end of transected major peripheral motor-sensory nerves is associated with the development of residual and/or PLP. Hence, the need for reoperation after amputation is high, with rates ranging from 40 to 50% [10]. Various surgical techniques based on neuroma resection and prevention of recurrence have been developed with varying results, including the transposition of proximal nerve stumps into surrounding tissues such as bone, vein, and muscle [9,11-14]. More recently, two novel surgical techniques for peripheral nerve reconstruction, targeted muscle reinnervation (TMR) and regenerative peripheral nerve interface (RPNI), have demonstrated lower rates of terminal neuroma formation when employed in patients to create neuromuscular interfaces for bionic prosthetic reconstruction (see below) [5,15,16].

Nerve reconstruction

Targeted muscle reinnervation (TMR) refers to the coaptation of the proximal nerve stump to a nearby motor branch of a previously denervated muscle to provide novel target organs for axonal regeneration and reinnervation following resection of a painful neuroma [4]. Ideally, nerves are size matched to avoid axonal escape and optimize nerve transfer (ingrowth). By giving these regenerating axons a novel target organ, the development of a neuroma may be prevented. In a retrospective, single-blinded randomized controlled trial, Dumanian et al. reported greater reductions in PLP scores after TMR compared to patients who received standard treatment with neuroma excision and burying the proximal nerve ending in adjacent muscle [16]. When performed at the time of amputation, patients who received TMR reported less PLP and neuroma-related pain [17]. In fact, primary TMR had a 3X greater chance of decreasing pain severity [17].

Another method of nerve reconstruction is RPNI. The creation of a RPNI involves implanting the transected nerve into a devascularized, denervated autologous skeletal free muscle graft to provide novel target organs for regenerating axons. Unlike TMR, the surgical technique for RPNI is technically simpler as it does not require microsurgery to coapt nerve endings. This method was initially described for better prosthetic control but was also found to significantly improve both stump pain and PLP. Rat models of RPNI after sciatic nerve transection [18] showed that RPNI significantly reduced behaviors associated with pain, nerve stump hyperplasia and fibrosis. Kubiak et al. performed a retrospective review of postoperative outcomes of 90 patients which showed that RPNI resulted in a lower incidence of both symptomatic neuromas and PLP compared with control patients [19]. While both TMR and RPNI clearly reduce pain, PAP is variable, and it is unclear which patients may benefit from these nerve reconstruction surgeries.

Peripheral nerve blockade

As an alternative to surgical management of PLP, peripheral nerve blocks were initially introduced to provide stump pain analgesia. Recent studies have shown that PNB can be used specifically for PLP. A multicentered randomized controlled trial [20] found that a continuous six-day ambulatory peripheral nerve block (PNB) resulted in a significant decrease in PLP four weeks after initiation. In some cases, relief lasted up to six months, however, nerve blocks did not provide long-term relief. Here, we aim to describe perioperative peripheral nerve blocks as a diagnostic tool for identifying symptomatic neuromas in patients who would benefit from the aforementioned surgical peripheral nerve reconstruction following major limb amputation.

## Materials and methods

Study design

A retrospective chart review was performed at the University of Maryland Medical Center on lower extremity amputees who underwent reconstructive nerve surgeries. The study was approved by the Institutional Review Board at the University of Maryland Medical Center (approval number: HP-00099041) and informed consent was waived.

Data collection

Patients 18 years or older who underwent prior major lower extremity amputation and subsequent reconstructive nerve surgeries (TMR and/or RPNI) between June 2021 and November 2023 were included in this study. Patients who did not receive a diagnostic nerve block before nerve reconstruction were excluded. Nerve blocks consisted of a single injection of lidocaine and/or bupivacaine to the tibial or common peroneal nerve. Data was collected retrospectively on demographics and clinical characteristics at initial and follow-up clinic visits as well as hospital admission for nerve reconstructive surgery.

Study outcomes

The primary outcome of this study was the frequency of PLP after nerve reconstruction relative to their preoperative PLP. Additional secondary outcomes included the use of medications for pain management (opioids, gabapentinoids, serotonin-norepinephrine reuptake inhibitors (SNRIs)) and the ability to ambulate with and without assistance. Ambulation with assistance included devices such as walkers and canes but excluded prosthetics. 

Statistical analysis

Descriptive statistics were used to analyze the means, standard deviations and frequencies. Fischer’s Exact test was used to calculate statistical significance. A p-value <0.05 was considered statistically significant. All calculations were performed using Microsoft Excel.

## Results

Demographic information

Ten patients were initially considered for this review. Six patients met the review criteria. Demographic information is presented in Table 1. The average age was 68 ± 8.47, with one female participant (16%). The majority (83%) of patients were on a statin and/or aspirin. All patients had undergone a below knee amputation (BKA). The average time between amputation and revision was almost two years (Mean: 22.35 months, Range: 4.60-40.53 months). The type of nerve repair surgery varied between patients, but the majority (83%) of patients underwent TMR. One patient underwent nerve repair with both TMR and RPNI. The average time from revision to last follow-up appointment was 15 months.

**Table 1:** Patient demography Demographic information of patients who received a peripheral nerve block (PNB) before nerve construction surgery (N=6). Continuous variables are expressed as mean (standard deviation, SD), and categorical/binary variables are expressed as N (%). TMR: Targeted muscle reinnervation; RPNI: Regenerative peripheral nerve interface; T2DM: Type 2 diabetes mellitus.

Variable	N=6
Age (mean, SD)	68.33 (8.47)
Sex (F) (n, %)	1 (16.66)
Antiplatelet use (n, %)	5 (83.33)
Anticoagulation use (n, %)	3 (50)
Statin use (n, %)	5 (83.33)
Positive smoking history (n, %)	5 (83.33)
History of T2DM (n, %)	3 (50)
Time to revision after amputation, months (mean, SD)	22.35 (15.43)
Follow-up time, months (mean, SD)	15 (11.05)
Nerve repair	
RPNI (%)	3 (50)
TMR (%)	5 (83.33)

Medical management of pain is displayed in Table 2. Gabapentinoids were the most used medication, followed by opioids and serotonin and norepinephrine reuptake inhibitors (SNRIs) such as duloxetine. There was a decrease in the number of patients requiring gabapentinoids after nerve revision surgery (83% vs 66%). There was no change in the frequency of opioid or SNRI use for pain management after revision surgery.

**Table 2:** : Pharmacological pain management The table lists the pain medications each patient received before amputation, after amputation, and after revision with targeted muscle reinnervation (TMR)/regenerative peripheral nerve interface (RPNI). SNRI: Serotonin-norepinephrine reuptake inhibitors; PRN: As needed; OD: Once a day; BID: Twice a day; TID: Thrice a day; Q4H: Every four hours.

Case	Medication Class	Dose		
		Pre-amputation	Post-amputation	Post-revision
1	Gabapentinoids	None	300mg gabapentin TID	None
	Opioids	5mg oxycodone Q3H PRN	15mg oxycodone Q6H PRN	5 mg oxycodone Q4H PRN
	SNRIs	None	None	None
2	Gabapentinoids	500mg gabapentin TID, pregabalin 75mg TID	600mg gabapentin TID	600mg gabapentin TID
	Opioids	50 mg tramadol Q6H PRN	None	50 mg tramdol Q6H PRN
	SNRIs	None	30mg duloxetine OD	None
3	Gabapentinoids	N/A	None	100mg gabapentin TID
	Opioids	N/A	None	5mg oxycodone Q4H PRN
	SNRIs	N/A	None	None
4	Gabapentinoids	N/A	800mg gabapentin BID, 150mg pregabalin BID	50 mg pregabalin TID
	Opioids	N/A	10mg oxycodone PRN	5mg oxycodone Q6H PRN
	SNRIs	N/A	None	None
5	Gabapentinoids	None	100mg gabapentin BID	300mg gabapentin BID
	Opioids	None	5-325mg oxycodone-acetaminophen Q6H PRN	2.5mg oxycodone Q4H PRN
	SNRIs	None	None	None
6	Gabapentinoids	75mg pregabalin OD	75mg pregabalin OD	None
	Opioids	None	15mg oxycodone Q4H PRN	5mg oxycodone Q4H PRN
	SNRIs	75mg venlafaxine OD	75mg venlafaxine OD	75mg venlafaxine OD, 20mg duloxetine OD

PLP after nerve reconstruction

**Figure 1:**
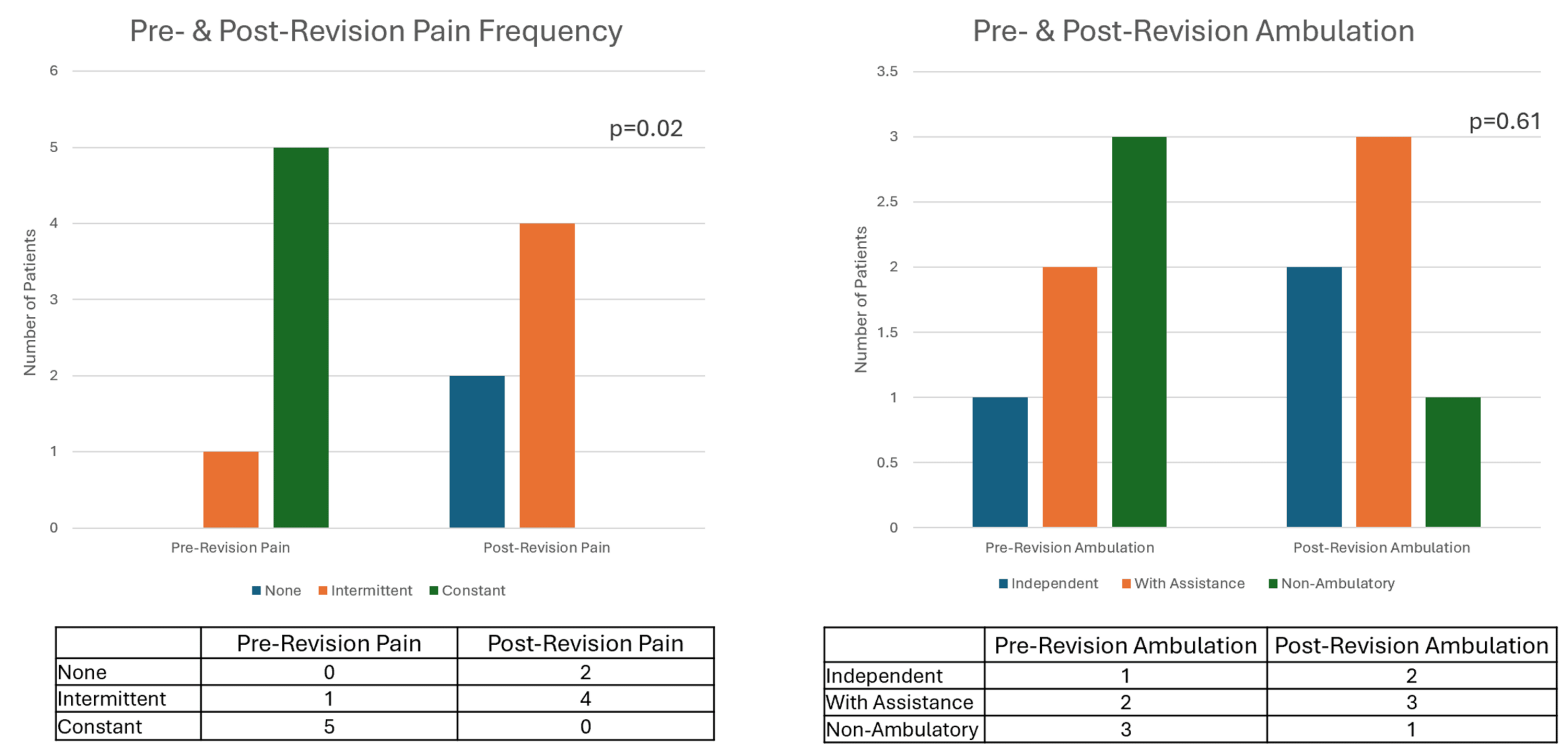
Prevalence of PLP and patient ability to ambulate before and after nerve reconstruction surgery The table lists the number of patients experiencing no pain, intermittent pain, or constant pain. Ambulation with assistance includes devices such as canes and walkers (excluding prosthetics). PLP: Phantom limb pain.

Prior to nerve revision surgery, most patients experienced chronic and constant PLP. There was a significant difference in the frequency of PAP after nerve reconstruction surgery (p=0.02). All patients experienced a reduction in PLP after reconstructive surgery with TMR and/or RPNI. No patients reported constant pain after reconstructive surgery, and two patients (33%) no longer reported PLP at all. The majority (66%, n=4) of patients experienced intermittent pain. Data on ambulation rates is also displayed in Table 3. While there was no significant difference in ambulation rates after nerve reconstruction surgery (p=0.61), ambulation improved following revision (50% vs 83%, n=3 vs 5), with half of the patients being able to ambulate with the assistance of devices such as canes or walkers (Figure 1).

Representative case report

An 81-year-old-man with a past medical history of hypertension, type 2 diabetes, and peripheral arterial disease who underwent a left BKA due to chronic nonhealing transmetatarsal amputation presented to the vascular clinic due to continuous phantom limb pain that significantly limited his lifestyle. He reported severe PLP attacks every 30-60 minutes. His pain would often wake him at night and limited ambulation. He attempted pharmaceutical treatment with high dose gabapentin to manage his symptoms.

To confirm the etiology of the pain, the patient underwent a PNB with 8 mL of Marcaine solution. He experienced significant pain relief for 6-7 hours. Hence, he was considered a good candidate for nerve reconstruction surgery. The patient underwent peripheral nerve repair using TMR for the superficial peroneal nerve, deep peroneal nerve and tibial nerve. The superficial peroneal nerve was coapted to the medial head of the gastrocnemius motor nerve, the deep peroneal nerve to the lateral head of the gastrocnemius muscle motor nerve, and the tibial nerve to the flexor hallucis longus muscle. Up to 20 months after the revision, he continued to report a significant improvement in PLP, with only two to three episodes daily. He is now able to sleep comfortably.

## Discussion

With the rate of major limb amputations rising, PAP is a significant source of pain that impacts ambulation and overall quality of life. Medical management of PAP is based on multimodal pharmacological and behavioral therapy with varying results [21]. Surgical management historically relied on neuroma resection with the burial of the proximal nerve stump into surrounding tissues. More recently, novel nerve reconstruction techniques such as TMR and RPNI have demonstrated promising results in clinical trials by preventing neuroma recurrence and development with significant symptomatic relief [16,17]. PNBs provide temporary pain relief but do not address the underlying pathology. Nevertheless, PNBs can be used to identify patients with symptomatic neuroma who may benefit from peripheral nerve reconstruction using TMR or RPNI. Here, we found that all patients who experienced a reduction in PAP, specifically PLP, after a diagnostic PNB also reported a reduction in PLP episodes after nerve reconstruction surgery. Patients presented in this study experienced immediate postoperative symptom relief and significant improvement in pain long term. While there was no significant statistical difference in ambulation levels after nerve reconstruction surgery, overall, all patients reported improved ambulation and quality of life. However, surgical intervention had little impact on medical management of PAP despite all patients reporting overall reductions in pain scores. This is likely due to underlying comorbidities such as diabetic neuropathy requiring pharmacological management. Larger studies on the impact of surgical PAP management on pharmacologic pain management are still warranted at this point.

The traumatic neuromas that form after limb amputation send erroneous inputs to the CNS, resulting in tract reorganization and sensitization that contributes to inappropriate signaling that is perceived as PAP [6,7,22-24]. These changes, which are dependent on peripheral nerve input, are reversible [6,20,22,25]. A trial by Birbaumer et al. found that blockage of the brachial plexus reversed cortical reorganization and eliminated the current experience of PLP [22]. PNBs with lidocaine and/or bupivacaine temporally prevent the initiation and conduction of action potentials to eliminate or reduce pain signaling. Nevertheless, PNBs do not address the underlying pathology and their use can be expanded beyond temporary symptomatic treatment. As supported by this data, PNBs can identify neuromas contributing to reversible PAP and potential nerve targets for TMR and RPNI. This is the first report to our knowledge that explores this function of PNBs in patients with major limb amputations due to vascular disease.

RPNI and TMR are ideally employed at the time of amputation to prevent neuroma formation and the development of PAP. However, these techniques have also proven effective at the time of revision surgery. As demonstrated by our study, PNBs can be used as a diagnostic tool to identify patients with neuropathic pain by providing temporary PLP relief and thereby facilitate the identification of adequate surgical candidates for RPNI and TMR procedures.

Limitations

One of the major limitations of this study is sample size. There are few surgeons who incorporate TMR and RPNI techniques during an amputation revision. Further cases including the use of these techniques are needed to better characterize the effectiveness of PNBs as a diagnostic tool.

## Conclusions

PAP is a complex spectrum of phenomena that impacts the quality of life of millions of patients. Recent studies have shown that primary nerve reconstruction surgeries such as TMR and RPNI significantly reduce the incidence of PAP. However, it is unclear which patients will benefit from these techniques years after amputation. In this retrospective study, all patients reported a reduction in PLP and improvement in ambulation after nerve reconstruction surgery. Here, we show that PNBs can be used as a diagnostic tool to identify patients with neuroma-associated PAP that will significantly benefit from surgical revisions with TMR or RPNI.
